# Effect of Perineal Urethrostomy on the Length of the Urethra of the Cat: A Cadaveric Study

**DOI:** 10.3390/ani13182810

**Published:** 2023-09-05

**Authors:** Anna Shipov, Inbar Israeli, Jean-Philippe Billet, Yoav Adam, Joshua Milgram

**Affiliations:** 1 Veterinary Teaching Hospital-Koret School of Veterinary Medicine, Faculty of Agriculture Food & Environment, Hebrew University Jerusalem, Rehovot 7610001, Israel; anna.shipov@mail.huji.ac.il (A.S.);; 2 Freelance Surgery, Tel Aviv 5800001, Israel; 3 Freelance Surgery, New York, NY 14925, USA; 4 Centre Hospitalier Veterinaire Atlantia, 44200 Nantes, France

**Keywords:** urethra, stricture, obstruction, landmarks

## Abstract

**Simple Summary:**

Perineal urethrostomy (PU) is a surgery performed in male cats suffering from recurrent urethral obstructions, which are common due to the small diameter of their distal urethra. The goal of surgery is to create a wider urethral opening by anastomosing the pelvic urethra to the skin. To achieve this, the penile and intrapelvic urethra are detached from the surrounding soft tissue and pulled caudally until the urethra at the level of the bulbourethral glands can be sutured to the skin without tension. It is unknown whether mobilization and traction of the penile and intrapelvic urethra result in translation or stretching of the tissues. Our aim was to characterize and quantify the effect of performing a PU on the location and length of the pelvic urethra. Ten feline cadavers were used. Markers were placed on the pelvic urethra, and radiographs were acquired before and after performing a PU. The distance of each marker from a predefined landmark/origin and the positions of the markers relative to one another were measured. Perineal urethrostomy resulted in significant caudal translation of the markers relative to the predefined landmark; however, it did not result in a change of the relative distances between the markers, suggesting that a caudal translation of the urethra, rather than urethral stretching, is the major component of urethral mobilization following perineal urethrostomy.

**Abstract:**

Perineal urethrostomy in cats is indicated for urethral pathologies located distal to the bulbourethral glands. The description of the bulbourethral glands as the cranial landmark when performing a PU is based on the increased urethral diameter at this location, rather than on an anatomical limitation. This suggests that urethral pathologies cranial to the bulbourethral glands could potentially be treated with PU. At present, the extent to which the pelvic urethra can be mobilized is unknown. Characterization and quantification of the effect of PU on the pelvic urethra is required prior to attempting to define the location of the pelvic urethra, cranial to the bulbourethral glands, which can be exteriorized when performing a PU. Our aim was to characterize and quantify the effect of performing a PU on the location and length of the pelvic urethra. Methods: Ten male feline cadavers were used, and four markers were placed on the pelvic urethra via a ventral approach to the peritoneal and pelvic cavities. Two orthogonal radiographic views were acquired prior and subsequent to performing a PU. The distance of each marker to a predefined landmark/origin and the distances of the markers relative to each other were measured on all radiographs. Results: PU resulted in significant caudal translation of the markers relative to the predefined landmark on all radiographic views; however, PU did not result in a significant change in the distances between the markers. Conclusions: Performing a PU results in caudal translation and minimal stretching of the mobilized pelvic urethra.

## 1. Introduction

Feline lower urinary tract diseases are very common and include idiopathic diseases (i.e., feline idiopathic cystitis), urolithiasis, urinary tract infections (UTI), urethral obstruction, and less frequently, neoplasia, urethral stricture, and anatomic abnormalities. The frequency of these diseases changes dramatically with age, with idiopathic feline urinary tract disease and obstruction being the most common in young cats but decreasing in frequency with age, while urinary tract infection and urolithiasis become much more common in middle-age and old cats [1].

Feline interstitial cystitis (FIC) is one of the most common diseases of the lower urinary system in young cats, with a recurrence rate of up to 65% in affected cats [2,3]. This is an inflammatory, noninfectious disorder of the lower urinary tract, and several risk factors have been identified, including living indoors, obesity, decreased activity, and consuming a dry diet [4]. Feline interstitial cystitis occurs in both males and females and is a self-limiting (few days) disease when it occurs as a sole pathology. However, one of its potential complications in male cats is urethral obstruction. The most common cause of urethral obstruction in male cats is urethral plugs, which are composed of proteinaceous material and crystals and account for approximately 60% of the urethral obstruction cases. Urethral plugs form due to the inflammatory process in the urinary bladder in FIC, which promotes urine protein leakage that consequently combines with urinary crystals. Less common causes for obstructions include uroliths, strictures or neoplasia. Obstruction of the penile urethra is a frequent in male cats and occurs predominantly due to the narrow distal portion of the urethra [5,6,7].

Long-term medical management is required to decrease the high recurrence rates (30–40%) of FIC and urethral obstruction [8]. Management includes appropriate dietary modification, increased water consumption, and decreased environmental stress using environmental enrichment techniques. Occasionally, in addition to the above, behavioral modification with or without medications is required. Male cats with recurrent episodes of urethral obstructions or cats with obstruction that cannot be resolved by catheterization (e.g., urethral strictures, trauma, or neoplasia) may benefit from surgical procedures which remove the affected urethra and create a new, permanent opening using the wider pelvic urethra [9].

The most commonly performed surgical procedure to address recurrent obstructions is the perineal urethrostomy (PU) described by Wilson and Harrison in 1971 [10]. This technique involves amputation of the narrow penile urethra and mobilization of the pelvic urethra until a urethra of sufficient diameter to create a new opening is identified. Mobilization of the pelvic urethra is considered to be sufficient when the mucosa of the urethra, at a location with increased diameter, can be sutured to the perineal skin without tension [10,11]. The urethral diameter at the bulbourethral glands is increased compared to the penile urethra, and when normal, is of sufficient diameter to perform a PU. The bulbourethral glands are located on the midline cranial to the insertion of the penile crura onto the penis and caudal to the ischiatic arch. The bulbourethral glands are the recommended cranial landmark when performing a perineal urethrostomy [7].

Most urethral pathologies occur in the distal urethra; however, damage to the urethra may occasionally be present cranial to the bulbourethral glands, especially in cases with iatrogenic damage due to repeat catheterizations [12,13]. In cases where the location of the pathology is suspected to be cranial to the bulbourethral glands, a preoperatively positive contrast urethrogram is recommended. In these cases, perineal urethrostomy may be less appropriate, and other techniques such as urethral resection and anastomosis, trans-pelvic urethrostomies, stent placement or even use of autogenous grafts, in cases of severe tissue damage, can be used [7,14,15]. To date, there are no clear guidelines as to which procedure is most appropriate when urethral pathology occurs cranial to the bulbourethral glands. In order to evaluate the potential to perform a PU for lesions cranial to the bulbourethral glands, a better understanding of the effect of PU on the pelvic urethra is required. The aim of this study was to characterize and quantify the effect of performing a PU on the location and length of the pelvic urethra.

## 2. Materials and Methods

This study was performed using 10 castrated, adult male cats, euthanized at the Veterinary Teaching Hospital for reasons unrelated to this study. Cats with urinary tract pathologies were excluded from the study. Cat cadavers were donated with a signed owner consent form. Cats were refrigerated (−5°) after euthanasia and were tested between 12 and 32 h after euthanasia.

### 2.1. Effect of Perineal Urethrostomy on the Pelvic Urethra

Cats were first placed in dorsal recumbency, the perineum and abdomen were clipped, and a purse string suture placed to close the anus. The pelvic and abdominal urethra were approached via the ventral midline. The peritoneal cavity was opened from the umbilicus to the pubis by incising the skin, subcutaneous tissue, and linea alba. The pelvic cavity was opened by extending the skin incision over the ventral aspect of the pelvis and dividing the left and right adductor muscles on the midline. The ventral aspect of the pelvis was then isolated and removed by cutting the rami of the pubic bones, at the level of the iliopubic eminences, and cutting the rami of the ischii at the caudal extent of the obturator foramen. The ventral aspect of the intrapelvic urethra was exposed by dividing the overlying fat on the midline. Care was taken to preserve the lateral and median ligaments of the bladder as well as all other peri-urethral tissue.

**Figure 1:**
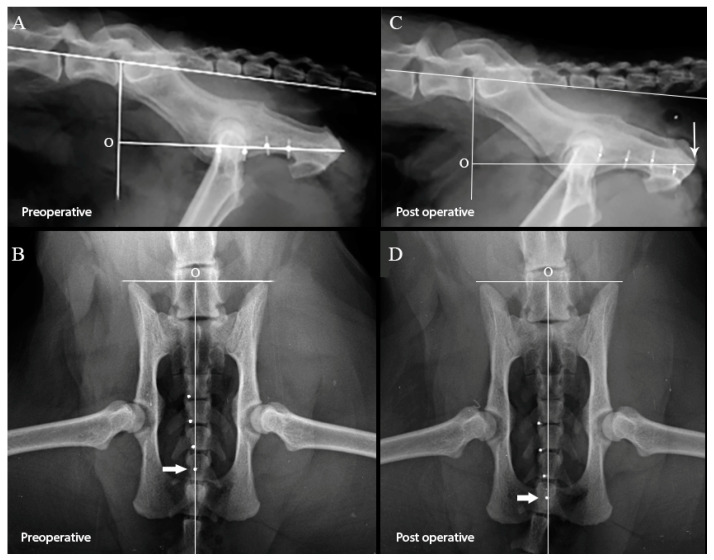
Preoperative lateral (**A**) and ventrodorsal (**B**) pelvic radiographs, and postoperative lateral (**C**) and ventrodorsal (**D**) pelvic radiographs of one of the cats used in this study. The lines used to define the predefined landmark/origin (marked “O”) have been placed on the radiographs. The location of the hemoclips has been enhanced by placing a white circle at the center of each hemoclip on the lateral radiographic views and directly over each hemoclip on the ventrodorsal radiographic views. Distances were measured between each consecutive marker and from the origin to each of the markers on all radiographs. The caudal translation of the markers as a result of performing a PU can be clearly seen, and the location of the caudal marker on the preoperative and postoperative ventrodorsal radiographic views are marked with the thick white arrow. The location of the medial aspect of the tuberosity of the ischium is marked with the thin white arrow.

The urethra was then marked at four locations with hemoclips, using bony landmarks to ensure consistency of placement. Locations were identified by holding a piece of suture material in contact with the bony landmarks on the right and left of the urethra and placing the hemoclip where the suture crossed the urethra. The cranial hemoclip (marker #1) was placed on the urethra at the level of the cranial aspect of the cranial osteotomy, and a second hemoclip (marker #2) was placed on the urethra at the level of the caudal aspect of the cranial osteotomy. The caudal hemoclip (marker #4) was placed on the urethra at the level of the caudal osteotomy. The distance between marker #2 and marker #4 was measured with a ruler, and an additional hemoclip (marker #3) was placed on the urethra half way between these two markers. The caudal abdomen and pelvis of the cat were then radiographed in two orthogonal views. The radiographs were assessed immediately and only included if the pelvis was well positioned and all four radiographic markers could be identified, unambiguously, on both radiographic views (Figure 1A,B).

A PU was then performed using the technique described by Wilson and Harrison [2,6]. All procedures were performed by two board-certified surgeons (Dip. ECVS).

After completion of the procedure, the cats were radiographed again using an identical technique. The radiographs were assessed immediately and identical inclusion criteria applied (Figure 1C,D).

A predefined landmark/origin served as the point from which measurements to each of the markers were taken. Bony landmarks, which could be reproduced consistently on all radiographs, were used to define the origin.

The location of the predefined landmark/origin as defined on the lateral radiographic view is illustrated in Figure 1A,C. A line was first drawn along the dorsal aspects of the vertebral bodies L5–L7, with a second intersecting line drawn at 100° to the first line, through the L7–S1 disc space. A third line, intersecting the second line at 90°, passing through the medial aspect of the ischial tuberosity, was the line along which all the measurements were taken, with the intersection of the second and third lines serving as the origin of the measurements.

The location of the predefined landmark/origin as defined on the ventrodorsal radiographic view, is illustrated in Figure 1B,D. A line was drawn along the cranial aspects of the ilial crests. A second line bisected the first line at 90° and was the line along which all the measurements were taken. The intersection of the lines served as the origin of the measurements.

The positions of the markers relative to the origin and relative to one another were measured using ImageJ software (https://imagej.nih.gov/ij/ access on 30 December 2019).

### 2.2. Statistical Methods

Quantitative variables were described as median and range. The distance between two consecutive markers before and after the procedure and the distance between each marker and the reference line before and after the procedure were compared using the Wilcoxon Signed Ranks Test; *p* < 0.05 was considered statistically significant. All analyses were performed with statistical software (SPSS 20.0 for Windows, Chicago, IL, USA).

## 3. Results

The median (range) distance between markers 1 and 2, markers 2 and 3, and markers 3 and 4, measured on both the lateral and VD radiographs, both pre and post PU are shown in Table 1. Performing a PU did not result in a significant change in the distance between any of the markers on either the lateral or VD radiographic views (Table 1). However, performing a PU led to a significant caudal translation of each marker relative to the predefined landmark/origin on all radiographic views (Table 1).

This indicates that mobilization of the urethra due to a PU procedure is caused mostly by translation. However, a borderline significant increase was seen between markers 2 and 3 on the VD radiographic view (9.2 mm vs. 9.8 mm, *p* = 0.052) and between markers 3 and 4 on the lateral radiographic view (9.0 mm vs. 9.9 mm, *p* = 0.059). An increased distance was also seen on the orthogonal radiographic view between these markers; however, the differences were not significant.

## 4. Discussion

Most cats with urethral obstruction are successfully managed using catheterization and short hospitalization. However, up to 58% of cases re-obstruct after initial discharge [8,16,17,18]. Perineal urethrostomy or other urine diversion techniques are considered as salvage procedures and as such, should only be performed when medical management has been exhausted and obstructions recur despite appropriate medical management. There are currently no guidelines as to the number or rate of recurrent obstructions at which surgical intervention is indicated, and the decision as to when to perform a PU is arbitrary. The high recurrence rate of urethral obstructions and the arbitrary nature of the decision to perform surgery make perineal ureterostomy one of the most common surgical procedures of the lower urinary tract.

Several surgical procedures have been described for cats with recurrent urethral obstruction; however, PU is the most commonly performed procedure in these cases. Key points for the success of a PU are accurate, tension-free apposition of the urethral mucosa to the skin of the perineum. Good surgical technique limits stricture formation during healing, resulting in a stoma with a functional diameter [19,20,21]. Urethral obstructions proximal to the bulbo-urethral glands may not be suitable for PU, and in these cases procedures such as trans-pelvic (ischial), sub-pubic, and pre-pubic urethrostomies are indicated [7,22,23,24]. Post-operative complications are common in all urethrostomy procedures and include but are not limited to urinary incontinence, recurrent infections, peristomal dermatitis, and subcutaneous urine leakage. Long-term complications, including urinary incontinence, are more commonly seen after pre-pubic urethrostomies [19,25,26,27]. Pre-pubic urethrostomy is the most commonly performed procedure for cranial urethral pathologies (where performing a PU is inappropriate), and long-term follow-up of these cases has been reported [23,28]. It has been shown that owner satisfaction and cats’ quality of life tend to be higher following PU when compared to pre-pubic urethrostomy. With these considerations in mind, it would be beneficial to define the most cranial location of urethral pathology where PU is indicated.

In this study, we characterized the movement of the pelvic urethra that occurs following PU, which, to the best of our knowledge, has not been previously described. We have shown that the pelvic urethra translates, rather than stretches, and the challenge now is to develop strategies to increase the translation of the urethra to the maximum allowed by the innervation and blood supply of the pelvic urethra. When performing a PU, it is paramount to perform adequate mobilization of the urethra to avoid complications such as dehiscence and stricture. This is achieved by blunt dissection of the ventral and lateral tissues which attach to the pelvic urethra. The dorsal attachments include both the innervation and the blood supply to the pelvic urethra, and minimal blunt dissection is performed in this location. Aggressive dissection of the tissues dorsal to the pelvic urethra may increase the translation of the pelvic urethra but may also damage the innervation of the pelvic urethra, resulting in postoperative incontinence [7,29]. It is possible that translation of the pelvic urethra will be increased by dissection of the tissues in this location. A detailed understanding of the location of the nerves and blood vessels dorsal to the pelvic urethra is required before dissection of the tissues in this location can be recommended. In addition, the length the nerves innervating the pelvic urethra will have to be determined as this is the factor which will determine the maximum extent to which the urethra can be translated caudally.

The external urethral sphincter which surrounds the urethra between the bulbourethral glands and the prostate is likely the reason that cats remain continent after PU. The sphincter is innervated by two branches of the pudendal nerve which reach the urethra at the level of the bulbourethral glands before continuing cranially toward the prostate gland [30]. Loss of continence as a complication of PU has been reported, and it is likely that in these cases, the innervation of the external urethral sphincter is compromised either by excessive translation or due to excessive undermining of the soft tissues surrounding the pelvic urethra [7,30].

This study demonstrates that most of the urethral movement during PU is due to translation, and it is possible that stretching of the urethra may be exploited to allow more cranial locations to be reached. Clinical recommendations as to how much the urethra can be stretched are also beyond the scope of this study; however, if excessive tension is encountered, a trans-pelvic urethrostomy can be performed in order to avoid complications associated with more proximal urethrostomies [31,32].

We limited our study to the intra-pelvic part of the urethra as we felt that this part of the urethra would show the greatest change when performing a PU. The effect of PU on the bladder and cranial urethra was not assessed in this study. However, one possible strategy for enabling the use of PU for more proximal pathologies is performing a caudal mobilization of the urinary bladder and proximal urethra to allow a greater translation of the intrapelvic urethra.

This study has several limitations. This is a cadaveric study; therefore, postmortem changes may have altered the properties of the urethral and surrounding tissues. In particular, contraction of the urethralis muscle with shortening of the urethra after isolation from the surrounding soft tissue may have been reduced and underestimated. The behavior of the urethra, both acutely and after healing, in the living animal undergoing PU was not evaluated and is beyond the scope of this study. The ventral approach to place markers may have resulted in excessive mobilization of urethra; however, when performing PU in the living animal, the ventral attachments of the urethra to the pelvis are freed as well, perhaps to a lesser extent, to allow mobilization of the pelvic urethra to the skin. A significant effort was made to limit dissection to structures attaching to the ventral aspect of the urinary system and maintain the median ligament of the bladder in all specimens to avoid additional mobilization of the urinary system. Additionally, since we have performed this mobilization during the initial surgical approach, thus it affected both pre- and post-PU radiographs; we feel that this did not act as a major limitation.

Measurement of the markers was performed along a line which we felt best approximated the location of the pelvic urethra. On the VD radiographic view, this line is a good approximation of the location of the urethra; however, on the lateral radiographic view it is likely that the line was ventral to the location of the urethra. The location of the line was selected to be parallel to the urethra and based on bony landmarks to ensure consistency. In addition, measurements were made along a single axis, and translation along other axes was not measured.

Although performing a PU did not cause a significant difference in the distance between consecutive markers, a borderline significant increase was seen between markers 2 and 3 on the VD radiographic view (9.2 mm vs. 9.8 mm, *p* = 0.052) and between markers 3 and 4 on the lateral radiographic view (9.0 mm vs. 9.9 mm, *p* = 0.059). An increased distance was also seen on the orthogonal radiographic view between these markers; however, the differences were not significant. This lack of significance might be due to type II error, and the true extent of urethral stretching should be further assessed. However, even if urethral stretching occurs, this appears to be negligible when considering the amount of translation. We speculate that if stretching of any part of the urethra was a significant clinical consequence of PU, then the incidence of stranguria in the post-operative period would be higher. The low incidence of this postoperative complication may support our findings that translation is responsible for the majority of the caudal movement of the urethra when performing a PU.

## 5. Conclusions

Perineal urethrostomy does not cause significant stretching of the intrapelvic urethra, and most of the movement of the pelvic urethra is a result of urethral translation.

## Figures and Tables

**Table 1:** The distance between markers prior and following PU as depicted in Figure 1.

Markers	Distance on Lateral View [Median (Range), mm]		*p* Value	Distance on Ventro-Dorsal View [Median (Range), mm]		*p* Value
	Pre PU	Post PU		Pre PU	Post PU	
Markers 1 and 2	8.5 (5.0–11.1)	8.3 (5.4–11.0)	0.610	8.7 (6.0–10.5)	8.5 (5.9–13.2)	0.310
Markers 2 and 3	9.1 (7.1–9.6)	9.2 (7.6–11.5)	0.230	9.2 (6.9–10.1)	9.8 (6.7–11.0)	0.052
Markers 3 and 4	9.0 (6.4–10.6)	9.9 (6.4–11.4)	0.059	9.5 (6.1–10.4)	9.4 (5.8–12.0)	0.553
Origin and marker 1	26.8 (19.2–31.9)	34.0 (18.7–37.6)	0.022	43.7 (39.3–51.8)	51.3 (43.2–58.1)	0.009
Origin and marker 2	34.7 (25.4–41.8)	41.77 (29.7–47.0)	0.037	53.8 (48.3–59.1)	61.1 (51.4–66.0)	0.005
Origin and marker 3	43.0 (34.6–51.6)	50.44 (37.3–58.5)	0.013	63.6 (55.9–67.9)	69.4 (61.2–75.9)	0.005
Origin and marker 4	49.6 (43.7–61.3)	58.7 (47.0–69.5)	0.009	71.4 (63.1–77.5)	77.6 (71.2–86.6)	0.005

## Data Availability

Data are available upon request.
